# Cellulose Acetate-Based Electrospun Materials with a Variety of Biological Potentials: Antibacterial, Antifungal and Anticancer

**DOI:** 10.3390/polym13101631

**Published:** 2021-05-18

**Authors:** Mariya Spasova, Nevena Manolova, Iliya Rashkov, Petya Tsekova, Ani Georgieva, Reneta Toshkova, Nadya Markova

**Affiliations:** 1 Laboratory of Bioactive Polymers, Institute of Polymers, Bulgarian Academy of Sciences, Akad. G. Bonchev St, bl. 103A, BG-1113 Sofia, Bulgaria; rashkov@polymer.bas.bg (I.R.); cekovapetya@polymer.bas.bg (P.T.); 2 Institute of Experimental Morphology, Pathology and Anthropology with Museum, Bulgarian Academy of Sciences, Akad. G. Bonchev St, bl. 25, BG-1113 Sofia, Bulgaria; rtoshkova@bas.bg (A.G.); ageorgieva@bas.bg (R.T.); 3 Institute of Microbiology, Bulgarian Academy of Sciences, Akad. G. Bonchev St, bl. 26, BG-1113 Sofia, Bulgaria; markn@bas.bg

**Keywords:** 5-chloro-8-hydroxyquinoline, cellulose acetate, electrospinning, antibacterial, antifungal and anticancer activities

## Abstract

Novel eco-friendly fibrous materials with complex activities from cellulose acetate and cellulose acetate/polyethylene glycol (CA,PEG) containing 5-chloro-8-hydroxyquinoline as a model drug were obtained by electrospinning. Several methods, including scanning electron microscopy, X-ray diffraction analysis, ultraviolet-visible spectroscopy, water contact angle measurements, and mechanical tests, were utilized to characterize the obtained materials. The incorporation of PEG into the fibers facilitated the drug release. The amounts of the released drug from CA/5-Cl8Q and CA,PEG/5-Cl8Q were 78 ± 3.38% and 86 ± 3.02%, respectively (for 175 min). The antibacterial and antifungal activities of the obtained materials were studied. The measured zones of inhibition of CA/5-Cl8Q and CA,PEG/5-Cl8Q mats were 4.0 ± 0.18 and 4.5 ± 0.2 cm against *S. aureus* and around 4.0 ± 0.15 and 4.1 ± 0.22 cm against *E. coli*, respectively. The complete inhibition of the *C. albicans* growth was detected. The cytotoxicity of the obtained mats was tested toward HeLa cancer cells, SH-4 melanoma skin cells, and mouse BALB/c 3T3 fibroblasts as well. The CA/5-Cl8Q and CA,PEG/5-Cl8Q materials exhibited anticancer activity and low normal cell toxicity. Thus, the obtained fibrous materials can be suitable candidates for wound dressing applications and for application in local cancer treatment.

## 1. Introduction

Cellulose acetate (CA) is derived from biorenewable resources and can be easily fabricated into diverse forms [1]. Moreover, it is a biodegradable and recyclable polymer with low cost and good physico-mechanical and barrier properties [2].

PEG is a water-soluble polymer with low toxicity, thus making it suitable for contact with living organisms. Moreover, PEG improves the solubilization of poor water-soluble drugs and could assist in their release [3]. Some of us have studied the effect of PEG incorporation on the release of 5-nitro-8-hydroxyquinoline (5N8Q) from the electrospun PLA/PEG fibers. The polyether incorporation was achieved by blending with or chemical grafting on PLA. The PEG incorporation method was proven to influence the release profile with the burst effect recorded for all PEG-containing mats [4].

8-Hydroxyquinoline and its derivatives manifest antibacterial, antifungal, and anticancer activities [5] and are of low toxicity to humans [6]. Therefore, these multi-action drugs have regained considerable interest in the last decade. They are suitable for application to the treatment of infections [7], cancer, tuberculosis, and other diseases [8,9].

Nowadays, there is a high demand for the development of multifunctional eco-friendly materials. Electrospinning is an attractive and feasible technique for the fabrication of polymer fibers with diameters down to the nano-scale range using the action of an external electric field imposed on a polymer solution or melt. The fiber morphology and diameters depend on the used polymer, solvent, salt addition, intrinsic properties of the polymer solution, processing conditions, and the ambient conditions. The adjustment of these parameters enabled the formation of thinner and uniform nanofibers [10].

The electrospun materials are promising candidates for different applications in medicine, pharmacy, cosmetics, agriculture, and industry [11]. The large specific surface area of the electrospun materials contributes to the enhancement of the release of the incorporated drug, to increasing its therapeutic effect, and to reducing the side effects. Therapeutic drugs, supplements, and wound healing accelerators that are actively involved in the healing process may be easily incorporated in the electrospun materials [12,13]. Furthermore, the COVID-19 pandemic has clearly shown the importance of developments in the fabrication of advanced materials. Electrospun materials with high drug loadings as oral delivery systems and advanced self-cleaning protective functional materials with excellent antibacterial and antiviral activities were obtained recently by electrospinning [14,15,16].

In our previous studies, it was shown that 8Q derivatives: potassium 5-nitro-8-hydroxyquinolate (K5N8Q) and 5-chloro-8-hydroxyquinolinol (5-Cl8Q), can be successfully incorporated into fibers from poly(lactic acid), poly(butylene succinate), cellulose acetate [17,18], poly(vinylidene fluoride), and poly(vinylidenefluoride-*co*-hexafluoropropylene) [19] in order to impart antibacterial and antifungal activities of the obtained fibrous materials. Up until now, no literature data are available on the incorporation of 5-Cl8Q in electrospun materials for local cancer treatment.

Cellulose acetate is suitable as a matrix for drug delivery, as it is a nontoxic, nonirritant, and biodegradable material with relatively good mechanical properties. However, the high hydrophobicity of some polymers restricts to a certain extent the application of such materials in the field of drug delivery, tissue engineering, etc. An easily achievable approach to modulate the hydrophilicity of the materials is the incorporation of a hydrophilic polymer in the spinning solution. Polyethylene glycols (PEGs) are highly hydrophilic polymers that are widely used in the biomedical field because of its set of properties, including the lack of toxicity and good biocompatibility. The electrospinning of mixed solutions of cellulose acetate, PEG, and biologically active substances will lead to the fabrication of perspective eco-friendly nano- and microfibrous materials with complex biological activities. The novelty of the present work consists of combining the advantageous properties of the cellulose acetate, PEG, and 8Q derivative by the electrospinning method, which is a promising strategy for the preparation of nanofibrous materials with complex activities suitable for local tumor treatment and wound dressing applications.

The present work aimed to study the possibility of preparing novel 8Q-containing nanofibrous mats by the one-step electrospinning of CA/5-Cl8Q and CA,PEG/5-Cl8Q solutions. The morphology and the chemical composition of the fibrous materials were characterized by scanning electron microscopy (SEM) and FTIR spectroscopy. The in vitro release profile of 5-Cl8Q from 5-Cl8Q-containing nanofibrous materials was determined. Antibacterial and antifungal analyses against Gram-positive bacteria (*S. aureus*), Gram-negative bacteria (*E. coli*), and fungi (*C. albicans*) were assessed. An evaluation of the in vitro antitumor activity of the CA-based and 5-Cl8Q-containing mats on the human cervical cancer cell line (HeLa), SH-4 melanoma skin cells, and non-cancer mouse BALB/c 3T3 fibroblasts using the MTT assay was also performed.

## 2. Materials and Methods

### 2.1. Materials

Cellulose acetate (CA, Aldrich, St. Louis, MO, USA) with a ${\bar{M}}_{n}$ of 30,000 g/mol and DS of 39.8%, polyethylene glycol (PEG, Fluka, Buchs, Switzerland) with Mr = 1900–2200 g/mol, and 5-chloro-8-hydroxyquinoline (5-Cl8Q, Sigma-Aldrich, Buchs, Switzerland) were used. Acetone (Sigma-Aldrich, Darmstadt, Germany) of analytical-grade purity was used. Dulbecco’s modified Eagle’s medium (DMEM) (Sigma-Aldrich, Darmstadt, Germany), enriched with fetal calf serum (FCS) (Gibco, Wien, Austria) and containing antibiotics (100 U/mL of penicillin, 0.1 mg/mL of streptomycin, LONZA, Cologne, Germany), was used. Trypsin–EDTA was supplied by FlowLab, Australia. 3-(4,5-Dimethylthiazol-2-yl)-2,5-diphenyltetrazolium bromide (MTT), ethidium bromide (EtBr), acridine orange (AO), and 4′,6-diamidino-2-phenylindole (DAPI) were purchased from Sigma–Aldrich, Darmstadt, Germany. The disposable consumables were supplied by Orange Scientific, Braine-l’Alleud, Belgium. HeLa human cervical cancer cells (ATCC, CCL-2), SH-4 (Homo sapiens skin melanoma) (ATCC, Rockville, MD, USA), and mouse BALB/3T3 clone A31 cell line (ATCC, CCL-163) were obtained from the American Type Cultures Collection (ATCC, Rockville, MD, USA). All chemicals used were of analytical grade and were used as received without any further purification.

### 2.2. Preparation of Electrospun Fibrous Mats

The optimal conditions for the successful preparation of fibrous materials from cellulose acetate (CA) and cellulose acetate/polyethylene glycol (CA,PEG) containing 5-chloro-8-hydroxyquinolinol (5-Cl8Q) were previously found [18]. The following fibrous mats were prepared by electrospinning: CA, CA/5-Cl8Q, CA,PEG, and CA,PEG/5-Cl8Q. For that purpose, different solutions were prepared in acetone/water 80/20 *v/v*: (i) CA, (ii) CA/5-Cl8Q, (iii) CA,PEG, and (iv) CA,PEG/5-Cl8Q. The total polymer concentration was 10 wt.% and the concentration of 5-Cl8Q was 10 wt.% in respect of the polymer(s) weight. Thus, prepared solutions were placed into a syringe (5 mL) equipped with a conical nozzle (gauge: 20GX1½″) connected to an electrode. The electrode was connected to a custom-made high-voltage power supply capable of generating positive DC voltages from 10 to 30 kV. The grounded rotating collector with a diameter of 45 mm was placed at a 15 cm distance from the needle tip, and the rotating speed was maintained at 1000 rpm. The spinning solution was delivered using an infusion pump (NE-300 Just InfusionTM Syringe Pump, New Era Pump Systems Inc., Farmingdale, NY, USA) at a controlled feed rate of 3 mL h^−1^, at a constant applied voltage (25 kV), at room temperature—21 °C, and at a relative humidity of 51%.

### 2.3. Characterization

The dynamic viscosity of the spinning solutions was measured using a Brookfield DV-II+ Pro programmable viscometer for the cone/plate option equipped with a sample thermostated cup and a cone spindle, at 25 ± 0.1 °C.

The morphology of the electrospun mats was evaluated by scanning electron microscopy (SEM). For this purpose, the mats were vacuum-coated with gold and were observed by a Jeol JSM-5510 SEM (Tokyo, Japan). The average fiber diameters, fiber distribution, and morphology were measured in terms of the criteria for the complex evaluation of electrospun mats reported elsewhere [20] using the Image J software program [21] by measuring at least 50 fibers from SEM images.

X-ray diffraction (XRD) analyses were performed using a computer-controlled D8 Bruker Advance diffractometer with filtered Cu Kα radiation and a LynxEye detector at room temperature. Data were collected in the 2θ range from 5.3° to 80° with a step of 0.02° and counting time of 1 s step^−1^. Diffracplus EVA using the ICDD-PDF2 Database was used for phase identification.

Attenuated total reflection Fourier-transform infrared (ATR-FTIR) spectroscopic analyses were performed using an IRAffinity-1 spectrophotometer (Shimadzu, Kyoto, Japan) equipped with a MIRacle ATR (diamond crystal; depth of penetration of the IR beam into the sample is ~2 μm) accessory (PIKE Technologies). The spectra were recorded from 4000 to 500 cm^−1^ with a spectral resolution of 4 cm^−1^ using a DLATGS detector equipped with a temperature controller. All spectra were corrected for H_2_O and CO_2_ using IRsolution internal software. All samples were dried under reduced pressure prior to analysis.

Static contact angle measurements were performed using a Krüss drop shape analysis system (DSA 10-MK2 model, Hamburg, Germany) at 20 ± 0.2 °C. A drop of deionized water (10 μL) controlled by a computer dosing system was placed onto the fibrous samples. Temporal images of the droplet were taken. The contact angles were calculated by computer analysis of the acquired images. The data were averages from 20 measurements on different areas of the mats’ surface.

Mechanical properties were evaluated via tensile measurements performed on the fibrous mats using a single column system for mechanical testing, INSTRON 3344, equipped with a loading cell of 50 N and Bluehill universal software. The stretching rate was 10 mm/min, the initial length between the clamps was 40 mm, and the room temperature was 21 °C. All samples were cut in the direction of collector rotation with dimensions of 20 × 60 mm^2^. A Digital Thickness Gauge FD 50 (Kafer GmbH, München, Germany) was used to determine the thickness of the fibrous materials. The average thickness was ca. 200 µm ± 20 nm. For the sake of statistical significance, 10 specimens of each sample were tested, after which the average values of Young’s modulus, the ultimate stress, and maximum deformation at break were determined.

The 5-Cl8Q content in the loaded mats was determined by dissolving a sample of 1 cm^2^ (~4 mg) in 10 mL of acetone/water = 80/20 *v*/*v*, and then measuring the absorbance by a DU 800 UV spectrophotometer (Beckman Coulter, Pasadena, CA, USA) at a wavelength of 255 nm.

The 5-Cl8Q release was studied in vitro at 37 °C in acetate buffer (CH_3_COONa/CH_3_COOH) containing lactic acid (acetate buffer/lactic acid = 96/4 *v*/*v*) at a pH of 3 and an ionic strength of 0.1. 5-Cl8Q-containing nanofibrous mats (4 mg) were immersed in 100 mL of buffer solution under stirring in a water bath (Julabo, Germany). The release kinetics was determined by withdrawing aliquots (2 mL) from the solution at the determined time intervals, adding back the same amount of fresh buffer, and recording the absorbance of the aliquots by a DU 800 UV–vis spectrophotometer (Beckman Coulter, CA, USA) at a wavelength of 255 nm. The amount of released 5-Cl8Q was calculated using calibration curves (correlation coefficient R = 0.999) for the mats in acetate buffer/lactic acid = 96/4 *v*/*v*, pH = 3, ionic strength 0.1. The data were average values from three measurements.

### 2.4. Antibacterial Assessment

The antibacterial and antimycotic activities of mats were monitored against the Gram-positive and Gram-negative bacteria *S. aureus* 749 and *E. coli* 3588 and against *C. albicans* 74. *S. aureus* 749, *E. coli* 3588, and *C. albicans* 74 were purchased from the National Bank for Industrial Microorganisms and Cell Cultures (NBIMCC, Sofia, Bulgaria). In order to measure the zones of inhibition, in vitro studies were performed using Tryptone glucose extract agar (DIFCO Laboratories, Detroit, MI, USA) solid medium. The surface of the solid agar was inoculated with a suspension of cell/fungi culture with a cell/fungi concentration of 1 × 10^5^ cells/mL, and on the surface of the agar, in each Petri dish, one mat was placed. The Petri dishes were incubated for 24 h at 37 °C and, subsequently, the zones of inhibition around the disks were measured. All tests were performed in triplicate. The average diameters of the zones of inhibition were determined using the ImageJ software based on 15 measurements in 15 different directions for each zone.

Moreover, the antimycotic activity of the fibrous mats against the fungi *C. albicans* 74 was assessed by a viable cell-counting method. Mats (4 mg) were UV-sterilized for 30 min on both sides and were then exposed to fungal suspension (2 mL) with a concentration of 10^6^ cells/mL^−1^ prepared in nutrient broth (Sigma-Aldrich) at 37 °C. Aliquots of 50 μL were taken at predetermined time intervals (4 and 24 h), and after ten-fold dilutions with sterile phosphate-buffered saline (PBS), they were placed on Petri dishes with nutrient agar (Sigma-Aldrich, Darmstadt, Germany). The plates were incubated at 37 °C for 24 h. The number of the surviving fungi was determined by counting the colony forming units (CFU) in triplicate for each experiment.

Evaluation of the adhesion of *C. albicans* 74 to the surface of the mats was performed by direct SEM observation with Jeol JSM-5510 (Jeol Ltd., Tokio, Japan). Briefly, the mats were incubated in 2.0 mL of broth culture of *C. albicans* 74 with a concentration of 10^6^ cell/mL for 72 h. Then, the mats were washed twice with PBS (pH 7.4) for the removal of nonadhered fungi. The adhered fungi on the surface of the mats were fixed by immersion of the mats in 2.5 wt.% glutaraldehyde solution in PBS at 4 °C for 5 h, and then carefully washed with PBS, freeze-dried, coated with gold, and observed by SEM.

### 2.5. MTT Cytotoxicity Assay

The effect of the different mats on the viability of HeLa cancer cells, SH-4 cells, and mouse BALB/c 3T3 fibroblasts was assessed by the MTT assay [22]. Briefly, the cells were trypsinized by 0.25% Trypsin-EDTA and were counted using a hemocytometer. Cells were transferred to a 96-well microtiter plate to ensure a concentration of 2 × 10^4^ cells/well. After overnight incubation at 37 °C in a humid atmosphere containing 5% CO_2_ to allow cells attachment, the culture medium was replaced and the cells were placed in contact with various mats (CA, CA/5-Cl8Q, CA,PEG, and CA,PEG/5-Cl8Q), preliminarily UV-sterilized for 30 min, and incubated for 24 and 48 h. HeLa cells, SH-4 cells, and BALB/c 3T3 fibroblasts incubated alone and in the presence of 5-Cl8Q were used as controls. Each variant was tested by five measurements. After culturing in the presence of mats, the HeLa, SH-4 cells, and BALB/c 3T3 cells were washed twice with PBS (pH 7.4) and further incubated with 100 μL of MTT working solution (Sigma Chemical) at 37 °C for 3 h; the supernatants were aspirated, and 100 μL of lysing solution (DMSO/ethanol 1:1) was added to each well to dissolve the resulting formazan. MTT assay reading was performed using an ELISA plate reader (TECAN, SunriseTM, Grodig/Salzburg, Austria). Cell viability was calculated as follows:

cell viability% = OD_570_(experimental)/OD_570_(control) × 100

### 2.6. Studying of Apoptotic Induction Using Dual Staining with AO and EtBr

The cell death of HeLa cancer cells, SH-4 cells, and mouse BALB/c 3T3 fibroblasts was quantified using acridine-orange (AO) and ethidium bromide (EtBr) double staining according to standard procedures [23].

HeLa cells, SH-4 cells, or mouse BALB/c 3T3 fibroblasts (1 × 10^5^ cells/well) were plated on glass lamellas, placed on the bottom of 24-well plates, at a concentration of 2 × 10^5^ cells mL^−1^, and incubated at 37 °C for 24 h in a CO_2_ incubator to form a monolayer. After that, the samples were placed in 24-well plates for a further 24 h of incubation. Then, the mats were removed, and glass lamellas were washed twice with phosphate-buffered saline (PBS, pH 7.4) to remove unattached cells, they were stained with AO and EtBr in the ratio of 1:1 (10 μg/mL), and they were then immediately examined with a fluorescence microscope (Leika DM 5000B, Wetzlar, Germany) within 30 min, before the fluorescence had started to fade away.

### 2.7. DAPI Staining

The nuclear morphology of the HeLa, SH-4 cells, and BALB/c 3T3 cells was assessed using 4′,6-diamidino-2-phenylindole (DAPI) staining. In brief, the cells (1 × 10^5^ cells/well) were cultivated on glass coverslips in the presence of the fibrous mats in 24-well tissue culture plates, in a CO_2_ incubator for 24 h. After incubation, the cells were fixed with 3% paraformaldehyde at room temperature. The fixed cells were then stained with a DAPI solution (1 μg/mL of DAPI in methanol) for 15 min at room temperature in the dark. The stained cells were coverslipped with 90% glycerol, and then the nuclear morphology was examined under a fluorescence microscope (Leika DM 5000B, Wetzlar, Germany).

### 2.8. Statistical Analysis

The data were displayed as means ± standard deviation (SD). To determine the statistical significance of the data, one-way analysis of variance (ANOVA) followed by Bonferroni’s post hoc test were performed. Values of * *p* < 0.05, ** *p* < 0.01, and *** *p* < 0.001 were considered significant.

## 3. Results and Discussion

### 3.1. Composition of Fibrous Materials: Morphology and Properties

Combining CA and PEG in the materials designed for biomedical applications have attracted interest, because CA and PEG are polymers of low toxicity. Moreover, the incorporation of the model drug 5-Cl8Q would impart to the prepared materials antibacterial, antifungal, and anticancer activities.

**Figure 1:**
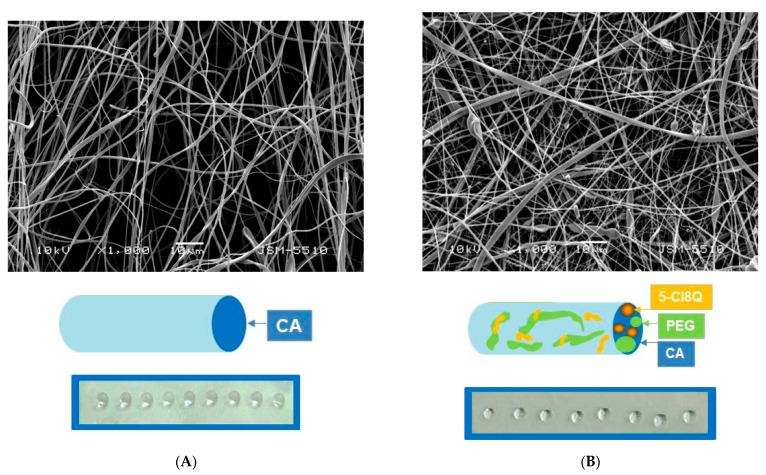

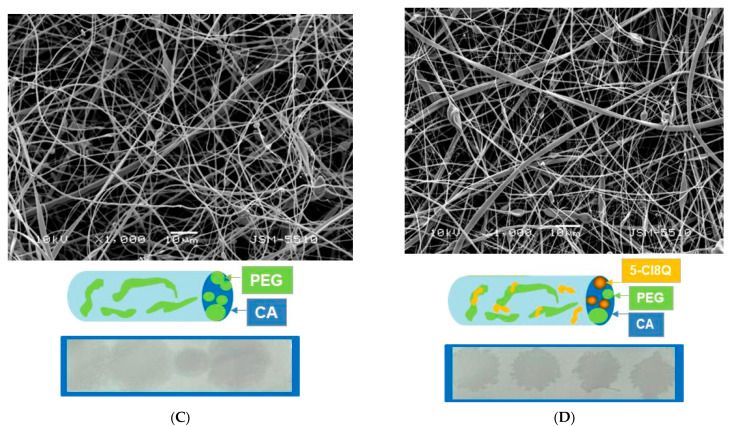
SEM micrographs, schematic representation of the cross-section of fibers and digital images of water droplets (10 µL) deposited on the mats’ surface of: CA (**A**); CA/5-Cl8Q (**B**); CA,PEG (**C**); CA,PEG/5-Cl8Q (**D**); SEM magnification ×1000.

A schematic representation of the cross-section of the fibers constituting the four types of fibrous mats (CA, CA/5-Cl8Q, CA,PEG, and CA,PEG/5-Cl8Q) is shown in Figure 1 (insets).

The dynamic viscosities of CA, CA/5-Cl8Q, CA,PEG, and CA,PEG/5-Cl8Q solutions were 184, 190, 91, and 108 cP, respectively. The values of the dynamic viscosity revealed that the incorporation of PEG into the CA solution resulted in a viscosity decrease owing to the low molecular weight of PEG. The incorporation of 5-Cl8Q in the solutions of CA and CA/PEG led to insignificant increases in dynamic viscosity values.

Figure 1 shows the SEM micrographs of the prepared fibrous mats. The fibers obtained from the CA solution with a concentration of 10 wt.% reproducibly resulted in the preparation of continuous defect-free fibers. The measured mean fiber diameters of CA and CA,PEG fibers were 780 ± 110 and 531 ± 100 nm, respectively (Figure 1A,B). The detected decrease in the diameter of CA,PEG fibers compared to CA was attributed to the decrease in the solution viscosity of the CA,PEG solution.

SEM images of the CA and CA,PEG fibrous mats, loaded with 5-Cl8Q, are presented in Figure 1B,D. The fiber diameters of these materials were 750 ± 120 and 446 ± 110 nm, respectively. It was found that the PEG addition reduced more significantly the fiber diameters than the addition of the model drug.

The possibility to design and control the surface and volume response is crucial for medical and pharmaceutical applications. For instance, the material surface that will uptake the liquid faster will also result in the faster release of the incorporated drugs, vitamins, bioactive compounds, etc. This will result in burst release during the first minutes or hours. In contrast, more hydrophobic material surfaces will result in an initial delay of the diffusion of the liquid media and a delay in drug release. Therefore, it is of importance to study the contact angle of the obtained fibrous materials in order to control the release behavior.

**Figure 2:**
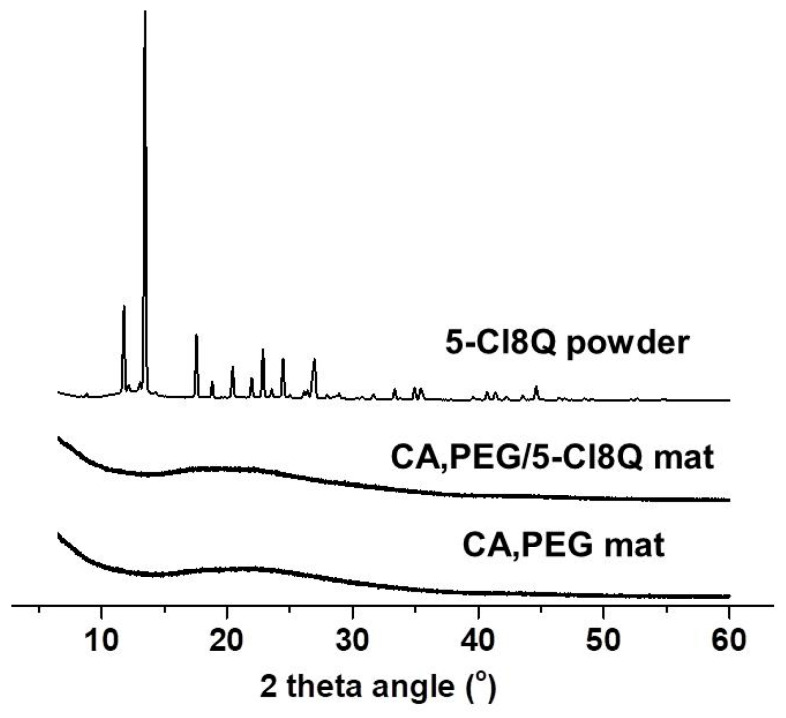
X-ray diffraction pattern of: CA,PEG and CA,PEG/5-Cl8Q fibrous mats and 5-Cl8Q powder.

In the present study, the water contact angles of the obtained materials were measured. Neat CA fibrous mats were hydrophobic with a water contact angle of 120.8° ± 3.0°. The water droplets were spherical and they preserved their shape for 2 h (Figure 1A). The measured contact angle values of CA/5-Cl8Q mats were 119.0 ± 3.2° and were close to those measured for the CA fibrous mats. The incorporation of water-soluble polymer, PEG, resulted in a significant decrease in the measured contact angle value. The values of the water contact angle of CA, PEG, and CA,PEG/5-Cl8Q mats were 0°, thus indicating complete wetting (Figure 1C,D). We have assumed that the fiber surface is mainly composed of CA; however, there are small areas rich in PEG that favor wetting and that lead to a significant reduction in the water contact angle. The incorporation of PEG even in small amounts resulted in the hydrophilization of the obtained fibrous materials and had a significant effect on the value of the water contact angle, which was 0°. It is known that electrospun materials can be used for drug loading and the amorphization of crystalline active pharmaceutical ingredients. Moreover, the electrospun fibrous materials demonstrate a reduction in the overall dose needed for the therapeutic activity, by improving the dissolution and bioavailability of the drugs [24]. Therefore, it was of interest to determine the state of the incorporated drug in the CA and CA,PEG fibers. The crystallinities of the obtained fibrous mats and 5-Cl8Q (powder) were analyzed by X-ray diffraction analysis (Figure 2). In the XRD patterns of 5-Cl8Q (powder), characteristic diffraction peaks of the drug were observed. These sharp diffraction peaks showed that the neat drug was highly crystalline. In contrast, the XRD analysis revealed that CA and CA,PEG mats were amorphous. Interestingly, the presence of an amorphous halo was detected for CA,/5-Cl8Q and CA,PEG/5-Cl8Q fibers as well. This finding revealed that 5-Cl8Q was in the amorphous state when incorporated into the fibers by electrospinning. The amorphization of 5-Cl8Q could be explained with the extremely rapid drying of the jet during the electrospinning, which hampers molecular motion. This observation indicates that electrospinning is an effective technique for the one-step incorporation of drugs/biologically active substances in fibrous materials where they are to be found in the amorphous state.

The CA and CA/5-Cl8Q fibrous mats were characterized by FTIR spectroscopy (Figure S1). The FTIR spectra of the CA fibrous materials showed bands characteristic of CA appearing at 1740 cm^−1^ for the C=O groups, at 1369 and 1226 cm^−1^ for the CH_3_ groups, and at 1037 cm^−1^ for the ether C–O–C groups [25]. In the FTIR spectra of CA/5-Cl8Q fibrous mats, in addition to the characteristic bands of CA, a new band appeared at 1500 cm^−1^, characteristic for the quinoline ring, proving the presence of the bioactive compound in the electrospun mat [26]. Clearly, no interaction between CA and 5-Cl8Q was detected on the FT-IR spectra of CA/5-Cl8Q fibrous materials.

**Figure 3:**
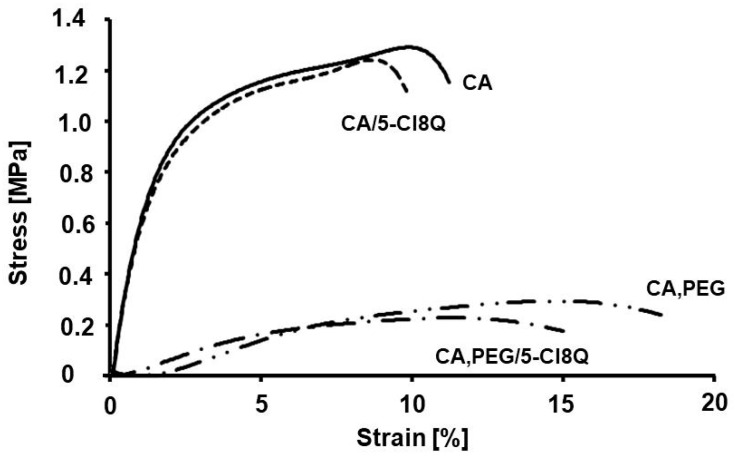
Stress–strain curves of CA, CA/5-Cl8Q, CA,PEG, and CA,PEG/5-Cl8Q mats.

Mechanical properties of the obtained fibrous materials were determined by using a single column system for mechanical testing. Optical images of the samples after testing were taken as well. The obtained stress–strain curves and the sample images are presented in Figure 3. The CA mats showed the highest values of tensile strength. Nevertheless, the detected values for the tensile strength and Young’s modulus of the CA fibrous mats were relatively low (c.a. 1.2 ± 0.15 MPa). This was most probably due to the semirigid backbone structure of cellulose acetate and to the fact that the fibers were loosely packed together. The incorporation of the model drug-5-Cl8Q did not considerably change the mechanical properties of the obtained materials. However, the incorporation of PEG with low molecular weight resulted in a significant decrease in the mechanical properties of the fibrous mats. The tensile strength of the CA,PEG fibrous materials was ca. 0.2 ± 0.05 MPa. This decrease in the mechanical characteristics could be due to the breaking of the structure of the continuous phase of CA. This finding is in accordance with literature data [27].

**Figure 4:**
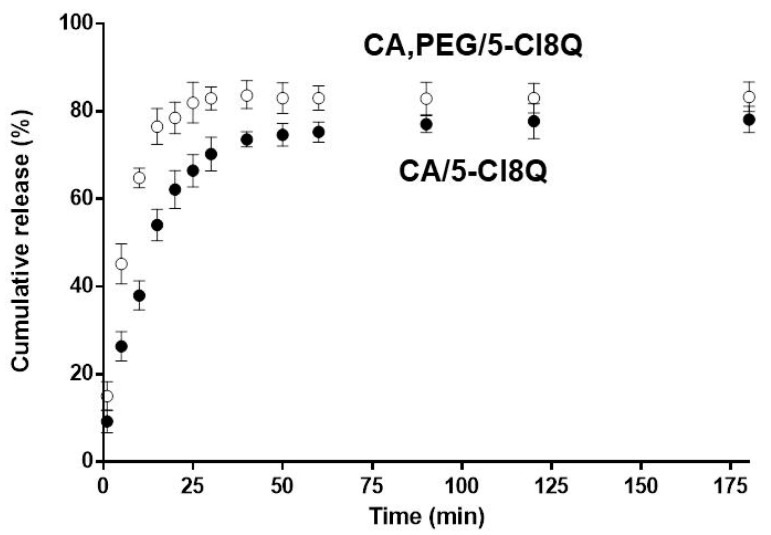
Cumulative release of the drug from the electrospun materials as a function of time. The error bars represent the standard deviation values. The results are presented as average values from three separate measurements; acetate buffer/lactic acid (96/4 *v*/*v*), pH 3, 37 °C, ionic strength 0.1; error bars indicate the standard deviation observed for three measurements.

The drug release from the electrospun mats of CA/5-Cl8Q and CA,PEG/5-Cl8Q was determined spectrophotometrically. The obtained results are presented in Figure 4. Initially, burst release was detected. After that, a gradual release was followed. The CA/5-Cl8Q fibrous mat released the drug at a smaller rate compared to the CA,PEG/5-Cl8Q mat. The amounts of the released drug from CA/5-Cl8Q and CA,PEG/5-Cl8Q were 78 ± 3.38% and 86 ± 3.02%, respectively (for 175 min). These differences in the release rate may be due to the different wettability of the fibers. The measured water contact angles for the CA/5-Cl8Q and CA,PEG/5-Cl8Q fibrous materials were 120° and 0°, respectively. The incorporated water-soluble polymer favors the penetration of the aqueous medium in the fibrous material and brings about the rapid drug release. It was found that the bioactive compound release was facilitated by PEG.

### 3.2. Antibacterial and Antimycotic Activities of the Fibrous Materials

Cellulose acetate is an eco-friendly material originating from a renewable resource. Moreover, it is biocompatible and biodegradable, and it possesses good mechanical properties. However, it does not possess intrinsic antibacterial and antifungal activities. Some of the recently used approaches to impart biological activities to cellulose derivatives involve the incorporation of metallic nanoparticles (such as silver, gold, copper, and titanium) (NP). Moreover, natural biopolymers could be used for the synthesis of NPs as well. These hybrid materials possess high antibacterial potentials against human pathogens [28].

8-Hydroxyquinoline derivatives display a broad range of biological activity such as antibacterial and antifungal activity. Among the derivatives, 5-Cl8Q showed a great inhibitory activity to *S. aureus* ATCC 25923 and to *E. coli* ATCC 25922 with a MIC of 89.09 μM. This compound exhibited activity against *C. albicans* ATCC with a MIC value of 178.17 μM as well [29].

**Figure 5:**
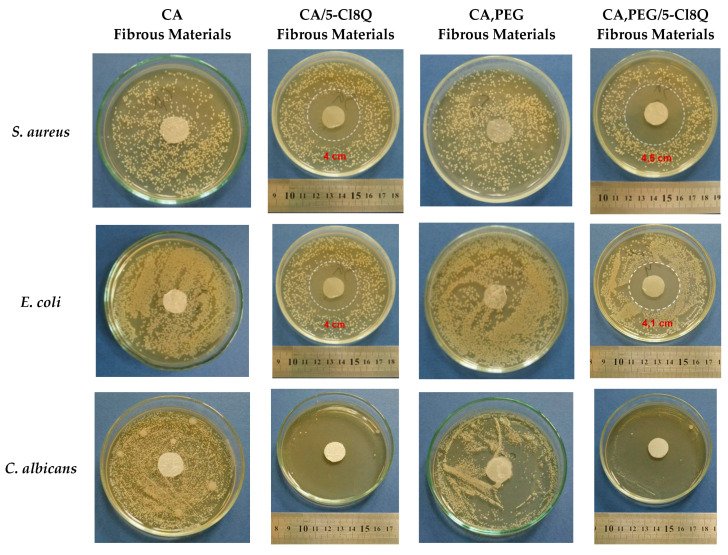
Digital images of the zones of inhibition against *S. aureus*, *E. coli*, and *C. albicans* after 24 h of contact of the fibrous materials (with a diameter of 17 mm) with bacterial and fungi cells. The mat type is indicated at the top of each column. The cell type is marked in the left of each row.

In the present study, the antibacterial and antimycotic activities of the electrospun mats were assessed by performing tests against Gram-positive bacteria *S. aureus*, Gram-negative bacteria *E. coli*, and fungus *C. albicans*. The results obtained by the determination of the zones of inhibition after 24 h of contact of the fibrous materials with the bacterial or fungal cells are shown in Figure 5. As expected, CA and CA,PEG mats did not exhibit any antibacterial and antifungal activities. Well-distinguished zones of inhibition of the bacterial and fungi cells growth were detected for the drug-containing fibrous materials. The diameters of the zones of inhibition of CA/5-Cl8Q and CA,PEG/5-Cl8Q mats were 4.0 ± 0.18 and 4.5 ± 0.2 cm against *S. aureus* and 4.0 ± 0.15 and 4.1 ± 0.22 cm against *E. coli*, respectively. The incorporation of 5-Cl8Q in the mats that were placed in contact with *C. albicans* resulted in the complete inhibition of the fungi growth. In contrast, the neat CA and CA,PEG mats did not alter the fungi growth and did not exhibit any antifungal activity. However, the incorporation of PEG resulted in the release to a higher rate of the biologically active substance, leading to the observation of wide zones of inhibition around the discs. The observation of zones of inhibition around all mats containing 5-Cl8Q is evidence that the incorporated drug imparts antibacterial and antifungal activities to the prepared novel fibrous materials.

**Figure 6:**
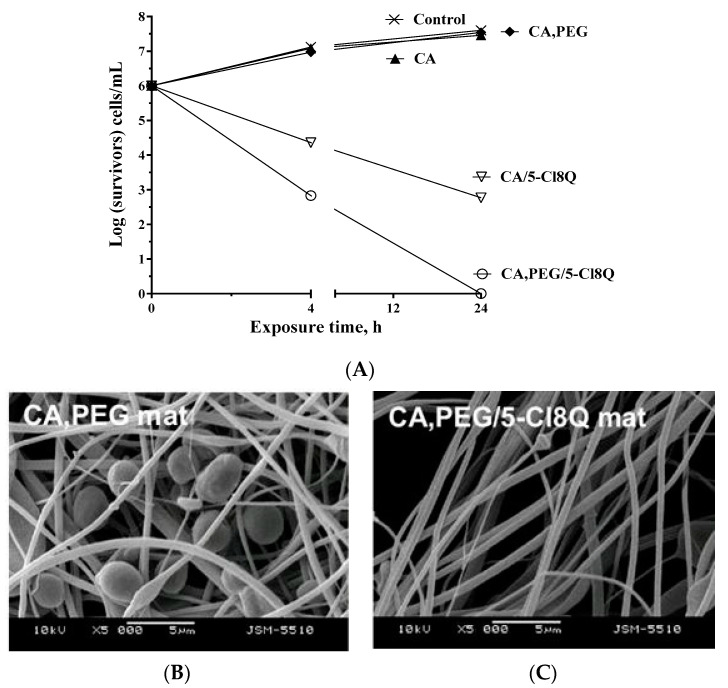
Logarithmic plot of the number of viable *C. albicans* cells versus the exposure time (**A**) and SEM micrographs of fibrous materials: CA,PEG (**B**) and CA,PEG/5-Cl8Q (**C**), that have been incubated in *C. albicans* cell culture (10^5^ cells/mL) for 24 h at 37 °C.

Moreover, the antimycotic activity of all materials against *C. albicans* was evaluated by counting the viable microorganisms that rested after the incubation of CA, CA/5-Cl8Q, CA,PEG, and CA,PEG/5-Cl8Q mats in fungi suspension for 4 and 24 h. The number of survived fungi was subsequently assessed by the plating and counting of CFU in solid medium. The log of the survived fungi vs. the exposure time for the electrospun materials is presented in Figure 6. For comparison, the growth of the *C. albicans* control was assessed as well. It was found that the control grew normally during the experiment.

Moreover, the fibrous mats of neat CA and CA,PEG did not affect the fungal growth, and after 24 h of contact, the number of cells reached 7 log. As seen from Figure 6A, a decrease in the number of the viable fungal cells was detected for the exposure time of 4 h for the fibrous materials containing 5-Cl8Q. The mats containing the model drug exhibited antifungal activity and, after 24 h of contact, a reduction in cell number was observed. Moreover, the CA,PEG/5-Cl8Q fibrous mats killed all *C. albicans* fungal cells at the 24th hour. This may be attributed to the PEG ingredient in the CA,PEG/5-Cl8Q materials that facilitates the release of the model drug and assists in manifesting its antifungal activity. On the other hand, the 5-Cl8Q incorporated into the hydrophobic CA mats released slowly. For CA/5-Cl8Q fibrous mats, a decrease in *C. albicans* titer by 3 log units was attained. It could be concluded that the polymers do not possess any intrinsic antibacterial activity. However, the incorporation of 5-Cl8Q imparted to the obtained materials significant antibacterial and antifungal efficiency.

In the present study, the adhesion of *C. albicans* cells onto the surface of the fibrous mats was monitored by SEM after incubation of the materials in a fungal suspension for 72 h at 37 °C. As seen from Figure 6B, a large number of *C. albicans* cells adhering to the surface of the fibrous CA,PEG mat was observed. The fungi retained their characteristic oval shape, morphology, and size (ca. 4 µm). On the contrary, the tendency of the prevention of the fungal adhesion and growth on the 5-Cl8Q-containing fibrous material was observed (Figure 6C) due to the high fungicidal effect of 5-Cl8Q incorporated in the fibrous mat. This observation, along with the results from the zones of inhibition and the number of viable cells, proved that the 5-Cl8Q-containing materials exhibit strong antibacterial and antifungal activities. Up to now, the exact mechanism of action of 5-Cl8Q in fungal cells is poorly understood. However, there are data in the literature suggesting that the mechanisms for antifungal activity of the 8-hydroxyquinoline derivatives occur mainly due to cell membrane damage. 8-Hydroxyquinoline and its derivatives can target ergosterol, which is a structural component of the membrane, forming transmembrane pores that lead to alterations in the membrane permeability with the loss of intracellular content and consequent fungal membrane disruption [30].

### 3.3. In Vitro Cytotoxicity Tests of the Fibrous Mats against HeLa Cells, SH-4 Cells, and BALB/c 3T3 Fibroblasts

**Figure 7:**
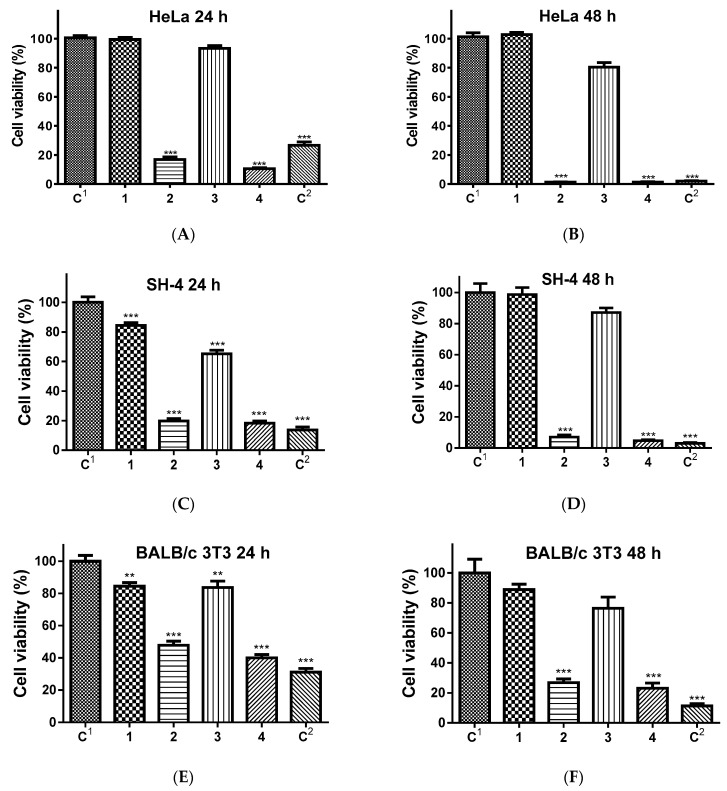
Cell viability of HeLa (**A**,**B**), SH-4 (**C**,**D**) and BALB/c 3T3 (**E**,**F**) cells tested by MTT method after (**A**,**C**,**E**) 24 h and (**B**,**D**,**F**) 48 h of incubation with different formulations: C1—untreated cells; 1—CA mat; 2—CA/5-Cl8Q mat; 3—CA,PEG mat; 4—CA,PEG/5-Cl8Q mat; C2—free 5-Cl8Q (100 µM/L); data are expressed as mean ± SD percent of viable cells and are representative of 5 experiments. ** *p* < 0.01, *** *p* < 0.001.

The MTT assay data of in vitro cytotoxicity experiments showed that all 5-Cl8Q-containing mats had pronounced anticancer activities against the human cancer cell lines, i.e., cervical cancer (HeLa) and melanoma skin cells (SH-4) (Figure 7A–D). The cell viability was not significantly affected by the nonloaded CA and CA,PEG mats, while the 5-Cl8Q-loaded CA/5-Cl8Q and CA,PEG/5-Cl8Q mats manifested a strong antiproliferative effect. The decrease in cell viability was higher at 48 h and was commensurate with the decrease observed at 48 h when treated with free 5-Cl8Q. After 48 h of incubation, the percentage of the viable HeLa cells placed in contact with CA/5-Cl8Q and CA,PEG/5-Cl8Q mats was ca. 1%. The percentages of viable SH-6 cells after 48 h were reduced to 7.0% and 4.7%, respectively. The higher antiproliferative activity of CA,PEG/5-Cl8Q mats compared to CA/5-Cl8Q mats might be attributed to the facilitated drug release from the hydrophilic CA,PEG mat. The nonmalignant BALB/c 3T3 cells were much less affected (Figure 7E,F), with 26.9% and 23.1% of viable cells after incubation in contact with CA/5-Cl8Q and CA,PEG/5-Cl8Q mats for 48 h, respectively. The 5-Cl8Q-containing mats exhibited a strong antiproliferative effect toward tumor cells while retaining the reduced toxicity against normal mouse fibroblasts.

### 3.4. Analysis of Cell Death by Staining Methods

**Figure 8:**
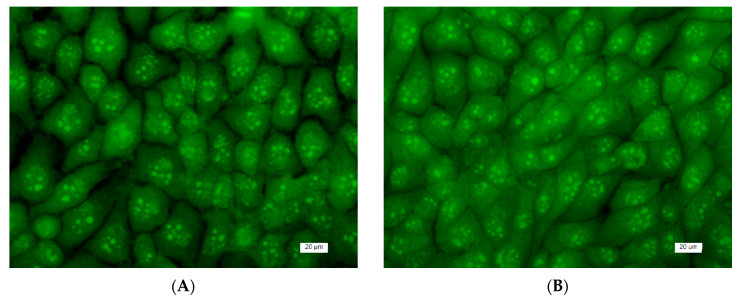

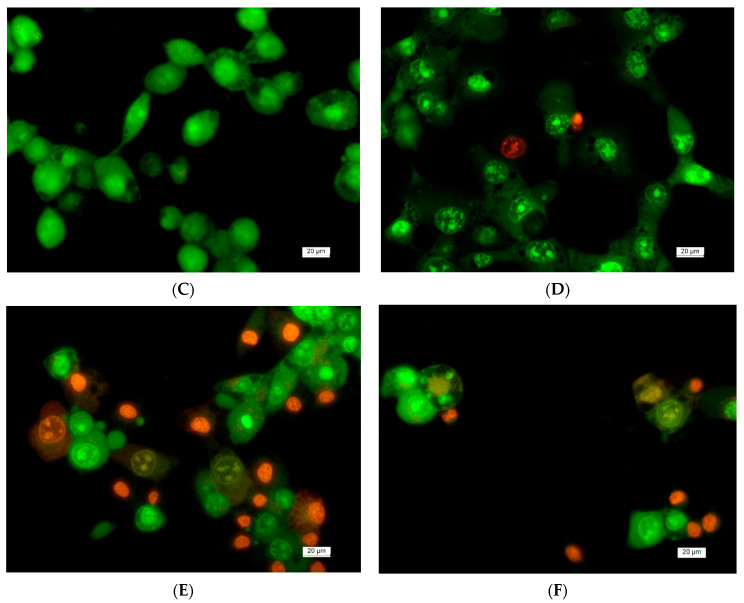
Fluorescence micrographs of AO and EtBr double-stained HeLa cells incubated for 24 h. (**A**) Untreated HeLa cells; (**B**) CA mat; (**C**) CA,PEG mat; (**D**) 5-Cl8Q solution; (**E**) CA/5-Cl8Q mat; (**F**) CA,PEG/5-Cl8Q mat. Live cells are shown in green and dead cells are shown in orange. Bar = 20 μm.

**Figure 9:**
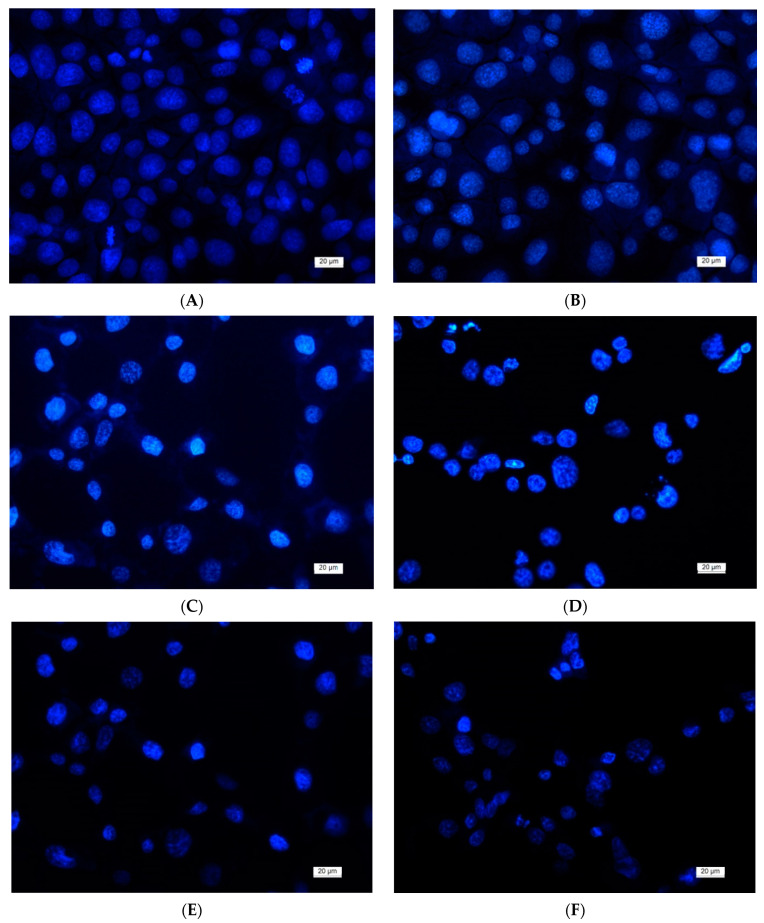
Fluorescence images of HeLa cells stained with DAPI: (**A**) untreated HeLa cells; (**B**) CA fibrous mat; (**C**) CA,PEG mat; (**D**) 5-Cl8Q solution; (**E**) CA/5-Cl8Q mat; and (**F**) CA,PEG/5-Cl8Q mat. Bar = 20 μm.

Apoptosis is a form of programmed cell death that occurs in multicellular organisms. Biochemical events lead to characteristic cell morphology changes and death. These changes include: blebbing, cell shrinkage, nuclear fragmentation, chromatin condensation, chromosomal DNA fragmentation, and global mRNA decay [31]. The morphological changes in apoptotic cells are clearly distinguishable at the light microscopic level. In order to identify the morphological changes in damaged tumor cells, the method of intravital double staining with fluorescent dyes (AO and EtBr) and DAPI was applied. For that purpose, HeLa cells were cultured for 24 h on CA, CA/5-Cl8Q, CA,PEG, and CA,PEG/5-Cl8Q materials, and they were stained using fluorescent dyes (AO and EtBr) (Figure 8). Untreated HeLa cells are characterized by a normal morphological structure with pale green nuclei and bright yellow-green nucleoli. The cells that were in contact with CA and CA,PEG mats had normal morphologies, as well. The number of HeLa cells that were in contact with CA,PEG was detected to be reduced (Figure 8C) in comparison with the cells of untreated cells and the cells cultured on the CA mat (Figure 8A,B). This can be explained by the fact that PEG exhibits cytostatic effects [32], but it is not cytotoxic [33]. As seen from Figure 9C, PEG did not have any negative effect on the cell growth and no dead cells were observed. In contrast to them, the contact with the fibrous mats containing 5Cl8Q resulted in a decrease in the number of HeLa cells and in the occurrence of morphological changes. Pale green and orange-red colored dead cells, the majority of which have the morphological characteristics of early or late apoptosis, were observed. The observed morphological changes included: cell rounding-up, cell shrinkage, condensation and aggregation of chromatin, and DNA fragmentation and formation of apoptotic bodies. A strong cytotoxic effect was observed in CA/5-Cl8Q and CA,PEG/5-Cl8Q mats (Figure 8D,F). A significant number of cells’ nuclei and cytoplasm were stained in orange-red.

It is known that 8-hydroxyquinoline derivatives show an anticancer effect and inhibit various cancer cell lines such as SK-OV-3, BEL-7404, HeLa229, T-24, MGC-803 [34], HuCCT1, and Huh28 [35], and they also have strong anti-leukemic activities [36]. Up until now, no data have been found in the literature concerning the anticancer effect of electrospun fibrous materials containing 5-Cl8Q toward HeLa cells. The results obtained in this study demonstrated that fibrous materials containing 5-Cl8Q induced morphological changes in HeLa cells and displayed good cytotoxicity toward the HeLa cell line. These results are in accordance with the data from the MTT test.

Furthermore, the morphological changes occurring in HeLa cells after 24 h of contact with mats were analyzed with DAPI staining as well. DAPI staining was used to observe changes in the nucleus of treated HeLa cells in vitro. Fluorescence microscopic images of the cells stained with DAPI are presented in Figure 9. The nuclei of the control (nontreated HeLa cells) had identical shapes and sizes with smooth edges and (uniformly distributed) unchanged chromatin (Figure 9A). Cell nuclei in different phases of mitosis were detected. HeLa cells that were placed in contact with the CA and CA,PEG mat showed a nuclei morphology identical to that of the control cells (Figure 9B,C).

After being exposed to CA/5Cl8Q and CA,PEG/5Cl8Q mats, the cancer cells displayed typical features of apoptosis, such as chromatin condensation, pyknosis of nuclei, fragmentation of the nuclei, and formation of apoptotic bodies (Figure 9E,F). These results indicate that the 5Cl8Q-containing mats induced cell death by apoptosis and are promising candidates as systems for local drug delivery in the treatment of cervical cancer. No data concerning the antitumor effect of cellulose acetate and polyethylene glycol electrospun mats containing a model drug 5Cl8Q against human cervical cancer HeLa cells were found in the literature. Based on the results obtained, it can be assumed that the strong cytotoxic effect of fibrous mats containing 5Cl8Q against HeLa cells is due to the fact that the polymer matrix facilitates the drug release, thus assisting to manifest its anticancer activity.

By applying fluorescence test methods, morphological changes in the nuclei of SH-4 tumor cells treated with CA/5-Cl8Q and CA,PEG/5-Cl8Q mats characteristic of cell death by apoptosis were detected (Supporting Information, Figures S2 and S3). The treatment of Balb/c 3T3 fibroblasts with 5-Cl8Q-containing electrospun mats and with free 5-Cl8Q led to morphological changes at the cell and nucleus levels, characteristic of early and late apoptosis, with changes being more pronounced after treatment with 5-Cl8Q-containing mats (Supporting Information, Figures S4 and S5). The results obtained from the fluorescence test methods support the data from the MTT tests for reduced cell viability when treated with 5-Cl8Q-containing materials.

## 4. Future Perspectives

There is an urgent demand in healthcare for the development of new technologies, devices, diagnostics, and advanced materials. However, advanced materials are complex to develop and manufacturing costs are high. Electrospinning offers a feasible, efficient, and cost-effective technique for the fabrication of 3D structures with specific design and high porosity. These materials resemble the architecture of the extracellular matrix and, therefore, could find applications in biomedicine as drug delivery systems, in regenerative medicine, in cell and tissue engineering, in filtration, etc. Nowadays, the number of patients with cancer is increasing worldwide. Novel, more effective materials in cancer treatment with minimized side effects, more biocompatibility, low toxicity, and controlled release properties are needed. In cancer treatment, electrospun nanofibrous materials possess many advantages such as drug encapsulation, which enable controlled and sustained drug release at the desired site of action with improved efficacy, as well as the possibility for surface modification and alignment. Although there is great progress in generating novel electrospun nanofibrous materials for in vitro cancer treatment, more research is needed for their safe preclinical and clinical applications. One of things future studies may consider is in vivo animal tests. This type of study will reveal the effectiveness of the obtained materials for the treatment of spontaneous and transgenic tumors in animals. Extensive in vitro, as well as in vivo, studies are essential, prior to clinical application in humans.

## 5. Conclusions

The one-step preparation of eco-friendly 5-chloro-8-hydroxyquinoline-containing nanofibrous materials was achieved by electrospinning. The obtained materials were morphologically, structurally, and chemically characterized. It was found out that the CA/5-Cl8Q fibrous mat released the drug at a smaller rate compared to the CA,PEG/5-Cl8Q mat. The amounts of the released drug from CA and CA,PEG were 78 ± 3.38% and 86 ± 3.02% respectively. The incorporation of 5-Cl8Q in the mats imparted considerable antibacterial and antifungal activities against *S. aureus* and *E. coli* bacteria and *C. albicans* fungi. Wide zones of inhibition were observed around the 5-Cl8Q-containing fibrous materials. Moreover, the 5-Cl8Q-containing mats displayed high antiproliferative activities against human cervical HeLa cancer and SH-4 melanoma skin cells while preserving the reduced toxicity against normal mouse BALB/c 3T3 fibroblasts. The induction of apoptosis is one of the major mechanisms of the antitumor activity of the prepared nanofibrous materials, which is verified by the performed fluorescence microscopy analyses. All these features indicate that the obtained novel fibrous mats are suitable candidates as antibacterial wound dressing materials, as well as for application in local cancer treatment.

## Supplementary Materials

The following are available online at https://www.mdpi.com/article/10.3390/polym13101631/s1, Figure S1: FTIR spectra of electrospun membranes, Figure S2: Fluorescence microscope images of AO/EB stained SH-4 human melanoma cells after treatment with 5Cl8Q-containing materials, Figure S3: Fluorescence microscope images of DAPI stained SH-4 human melanoma cells after treatment with 5Cl8Q-containing materials. Figure S4: Fluorescence microscopic images of AO/EB stained Balb/c3T3 mouse embryo fibroblasts after treatment with 5Cl8Q-containing materials, Figure S5: Fluorescence microscope images of DAPI stained Balb/c3T3 mouse embryo fibroblasts after treatment with 5Cl8Q-containing materials.
