# Supplementary material for: Cellulose Acetate-Based Electrospun Materials with a Variety of Biological Potentials: Antibacterial, Antifungal and Anticancer
Source: Polymers (Basel). 2021 May 18;13(10):1631. doi: 10.3390/polym13101631 (PMC8157284; doi:10.3390/polym13101631)
Supplement: Supplementary file 1 [file polymers-13-01631-s001.zip › polymers-1231518-supplementary.pdf]

Supporting Information

Cellulose acetate based electrospun materials with variety of biological potential: antibacterial antifungal and anticancer

Mariya Spasova[1]*, Nevena Manolova[1]*, Iliya Rashkov[1], Petya Tsekova[1], Ani Georgieva[2], Reneta Toshkova[2] and Nadya Markova[3]

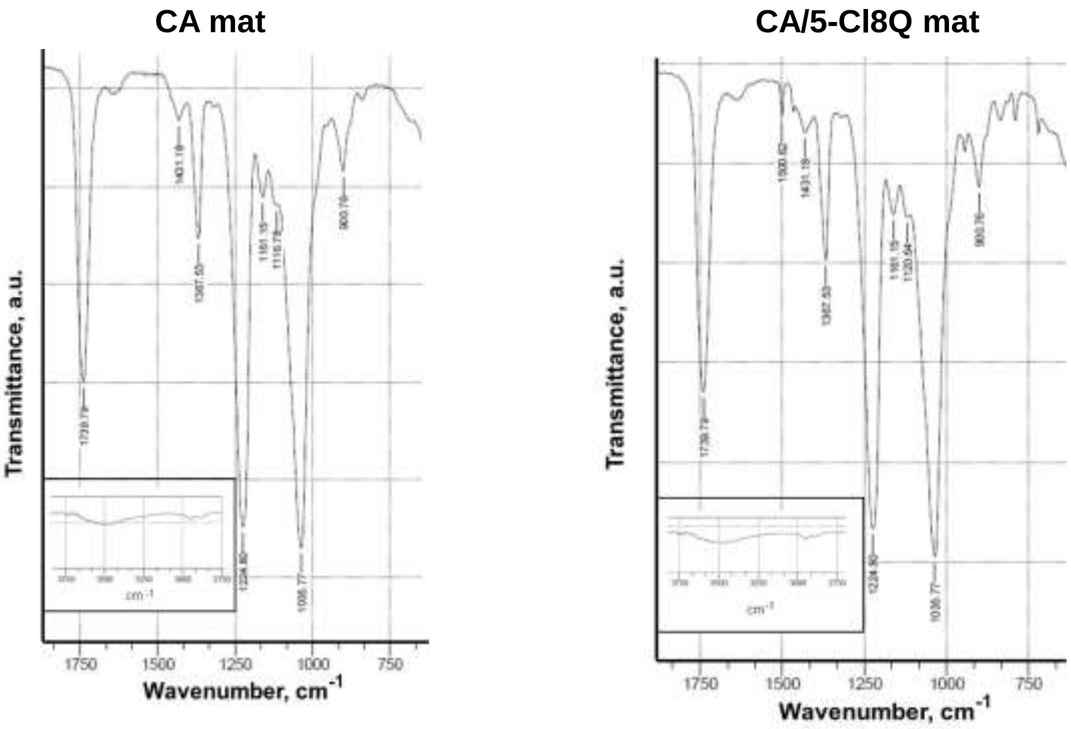

a b

Figure S1. FTIR spectra of electrospun membranes of: (a) CA and (b) CA/5-Cl8Q.

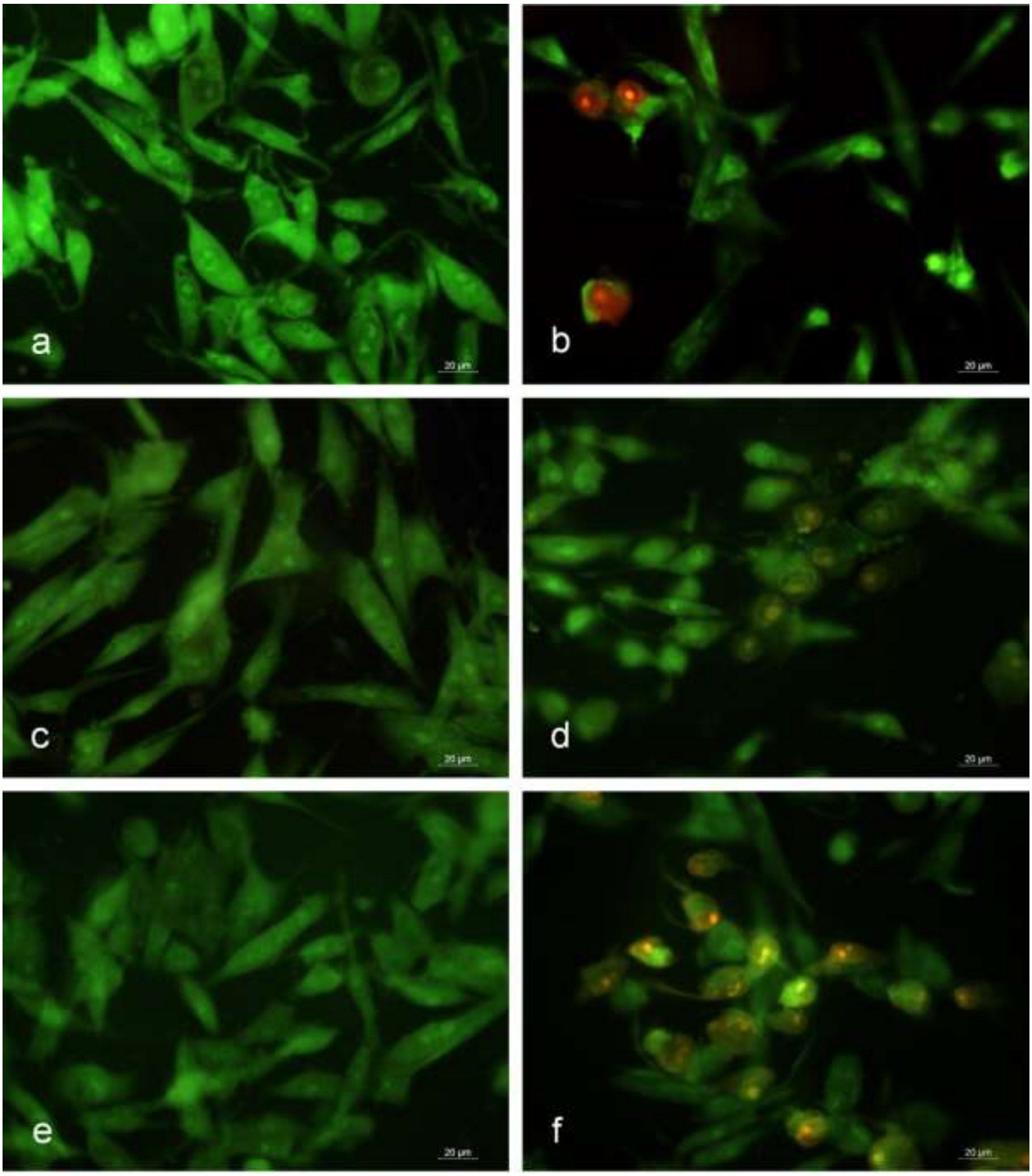

Figure S2. Fluorescence microscope images of AO/EB stained SH-4 human melanoma cells after treatment with 5Cl8Q-containing materials a) untreated control; b) 5-Cl8Q; c) CA mat; d) CA/5-Cl8Q mat; e) CA,PEG mat; f) CA,PEG/5-Cl8Q mat. Bar = 20 μm.

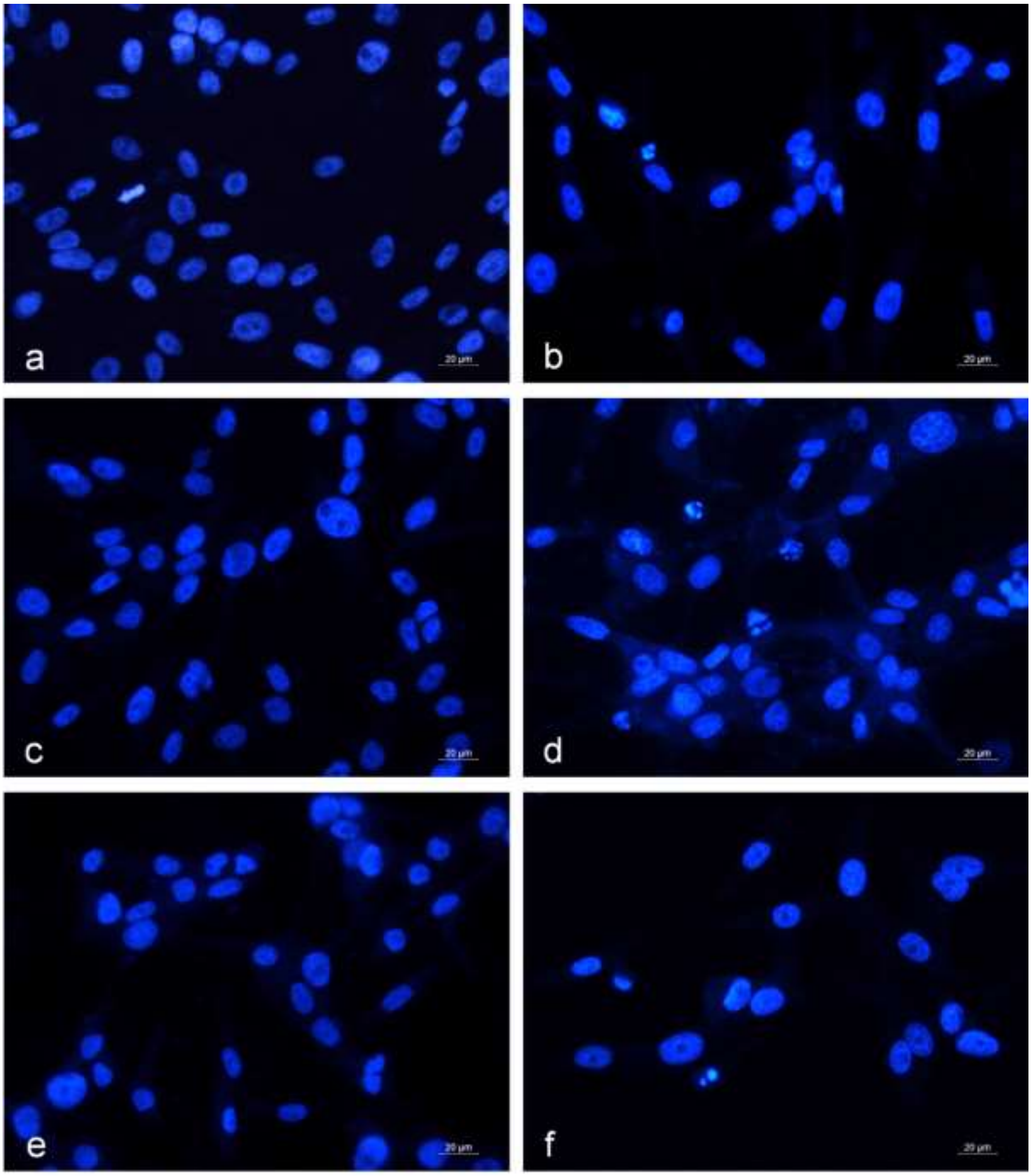

Figure S3. Fluorescence microscope images of DAPI stained SH-4 human melanoma cells after treatment with 5Cl8Q-containing materials a) untreated control; b) 5-Cl8Q; c) CA mat; d) CA/5-Cl8Q mat; e) CA,PEG mat; f) CA,PEG/5-Cl8Q mat. Bar = 20 μm.

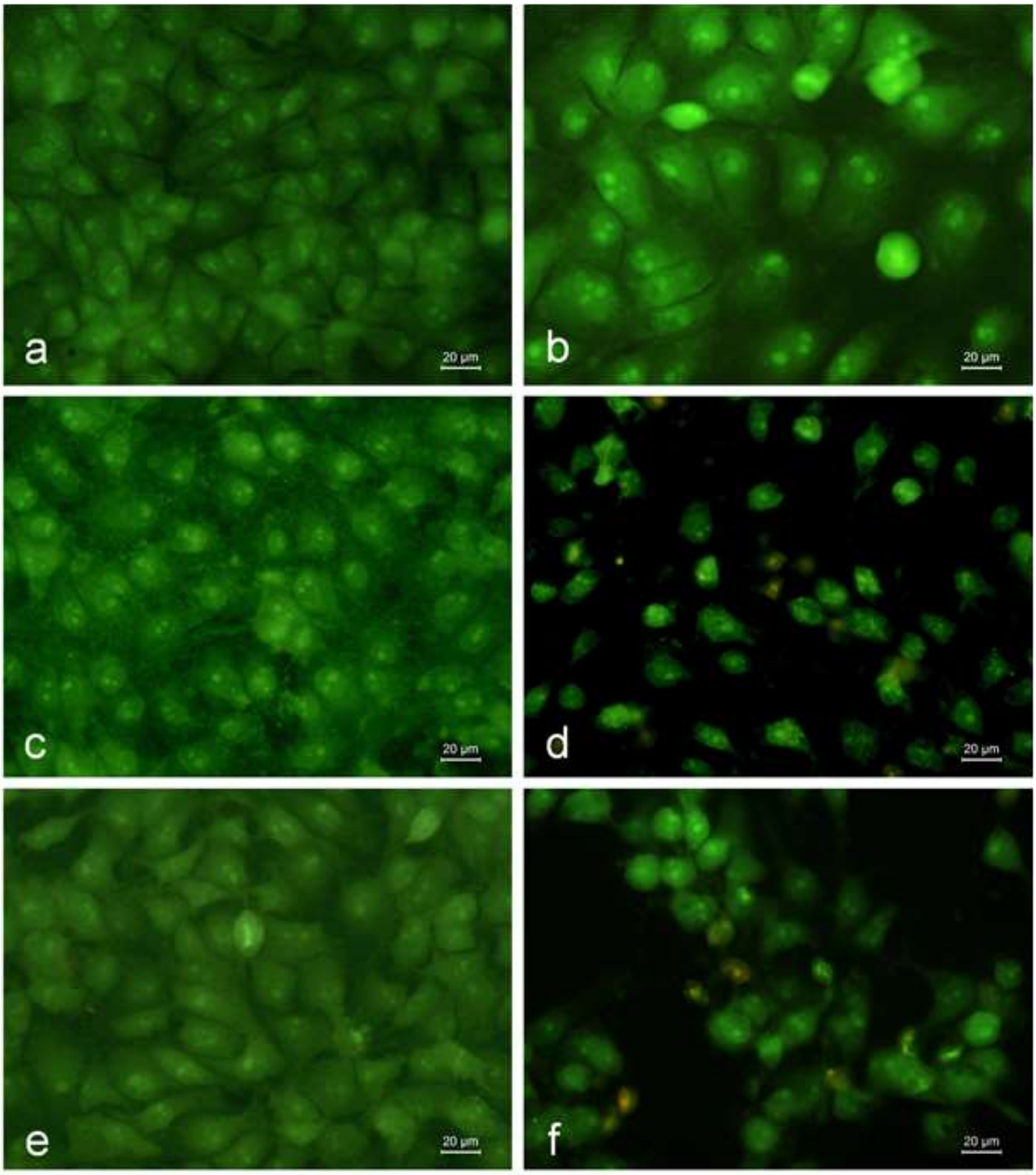

Figure S4. Fluorescence microscopic images of AO/EB stained Balb/c3T3 mouse embryo fibroblasts after treatment with 5Cl8Q-containing materials: a) untreated control; b) 5-Cl8Q; c) CA mat; d) CA/5-Cl8Q mat; e) CA,PEG mat; f) CA,PEG/5-Cl8Q mat. Bar = 20 μm.

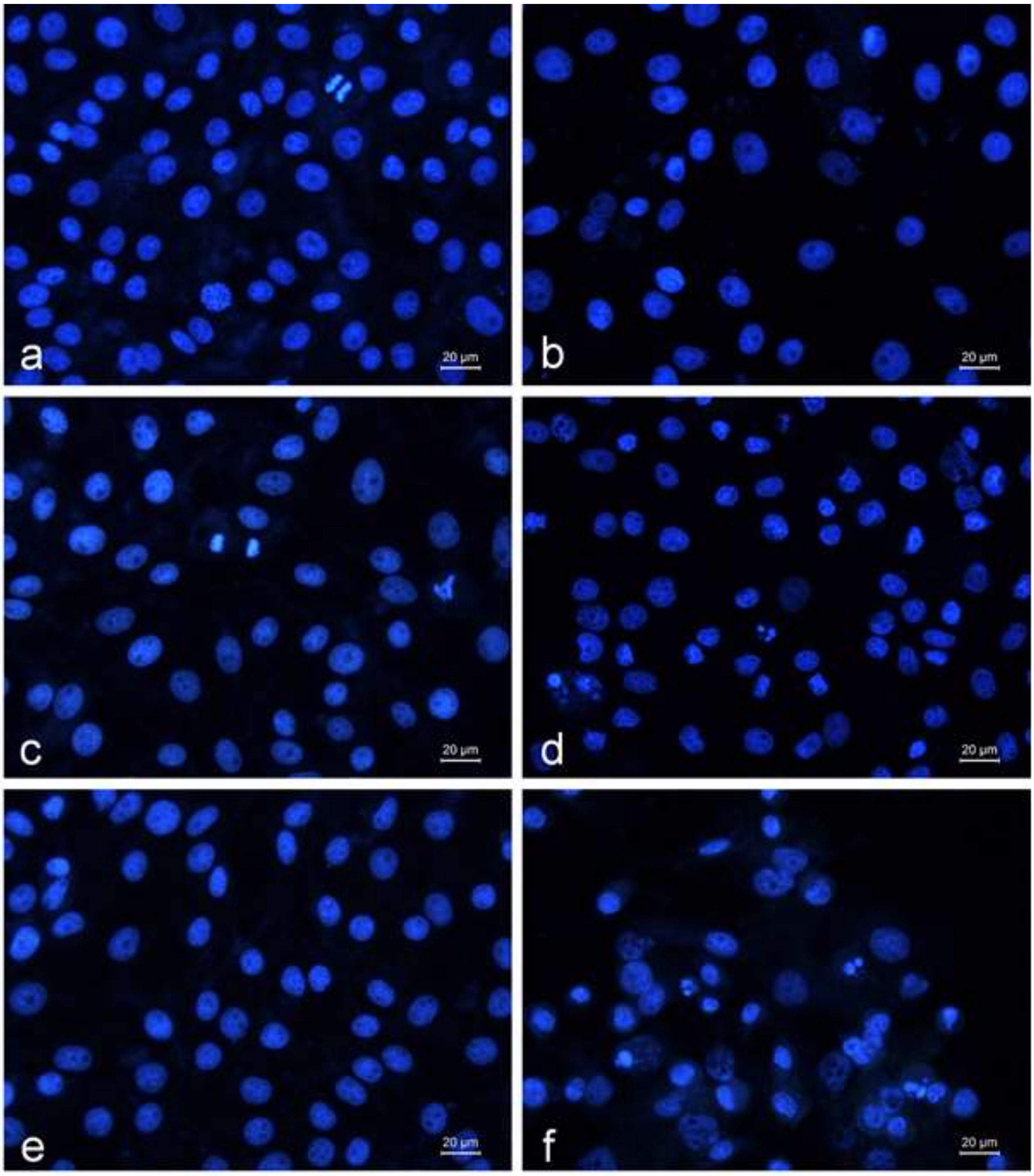

Figure S5. Fluorescence microscope images of DAPI stained Balb/c3T3 mouse embryo fibroblasts after treatment with 5Cl8Q-containing materials: a) untreated control; b) 5-Cl8Q; c) CA mat; d) CA/5-Cl8Q mat; e) CA,PEG mat; f) CA,PEG/5-Cl8Q mat. Bar = 20 µm.
